# Progressive brain abnormalities in schizophrenia across different illness periods: a structural and functional MRI study

**DOI:** 10.1038/s41537-022-00328-7

**Published:** 2023-01-05

**Authors:** Chen-Lan Shen, Shih-Jen Tsai, Ching-Po Lin, Albert C. Yang

**Affiliations:** 1 Institute of Brain Science, National Yang Ming Chiao Tung University, Taipei, Taiwan; 2 Department of General Psychiatry, Tsao-Tun Psychiatric Center, Ministry of Health and Welfare, Nantou, Taiwan; 3 Department of Psychiatry, Taipei Veterans General Hospital, Taipei, Taiwan; 4 Institute of Neural Science, National Yang Ming Chiao Tung University, Taipei, Taiwan; 5 Department of Medical Research, Taipei Veterans General Hospital, Taipei, Taiwan

**Keywords:** Schizophrenia, Biomarkers

## Abstract

Schizophrenia is a chronic brain disorder, and neuroimaging abnormalities have been reported in different stages of the illness for decades. However, when and how these brain abnormalities occur and evolve remains undetermined. We hypothesized structural and functional brain abnormalities progress throughout the illness course at different rates in schizophrenia. A total of 115 patients with schizophrenia were recruited and stratified into three groups of different illness periods: 5-year group (illness duration: ≤5 years), 15-year group (illness duration: 12–18 years), and 25-year group (illness duration: ≥25 years); 230 healthy controls were matched by age and sex to the three groups, respectively. All participants underwent resting-state MRI scanning. Each group of patients with schizophrenia was compared with the corresponding controls in terms of voxel-based morphometry (VBM), fractional anisotropy (FA), global functional connectivity density (gFCD), and sample entropy (SampEn) abnormalities. In the 5-year group we observed only SampEn abnormalities in the putamen. In the 15-year group, we observed VBM abnormalities in the insula and cingulate gyrus and gFCD abnormalities in the temporal cortex. In the 25-year group, we observed FA abnormalities in nearly all white matter tracts, and additional VBM and gFCD abnormalities in the frontal cortex and cerebellum. By using two structural and two functional MRI analysis methods, we demonstrated that individual functional abnormalities occur in limited brain areas initially, functional connectivity and gray matter density abnormalities ensue later in wider brain areas, and structural connectivity abnormalities involving almost all white matter tracts emerge in the third decade of the course in schizophrenia.

## Introduction

Since its original designation as “dementia precox” by Kraepelin in the 1900s, schizophrenia has been recognized as a chronic and deteriorating mental illness^1^. Computed tomography (CT) and magnetic resonance imaging (MRI) have also been reported to reveal structural and functional brain abnormalities in patients with schizophrenia^2,3^. However, whether these brain imaging abnormalities remain stationary or become progressive throughout the course of the illness has yet to be determined^4,5^.

Among structural brain abnormalities in schizophrenia, gray matter density change is the most commonly studied one, usually using voxel-based morphometry (VBM). Compared with that of healthy controls, gray matter density was reduced in some brain regions in patients with recent-onset schizophrenia^6^. In chronic schizophrenia, gray matter density reduction was observed in extensive brain regions^7^. In a recent review article, Howes et al. concluded gray matter density is lower relative to controls in a network of regions, including the bilateral insular cortex, anterior cingulate gyrus, left parahippocampal gyrus, middle frontal gyrus, postcentral gyrus, and the thalamus and the abnormalities progress from first episode to chronic stage in schizophrenia^8^. However, most studies have recruited patients with chronic schizophrenia lasting less than 20 years of ill duration, and the illness’s progression beyond this point has rarely been investigated^9,10^.

Diffusion tensor imaging (DTI) has been used to assess white matter abnormalities with fractional anisotropy (FA) serving as an index of white matter tract integrity. FA reflects the combined results of diffusion contributed by fiber arrangements, the degree of myelination, and axonal integrity and has been reported to decrease in patients with schizophrenia. A few studies have investigated FA progression throughout the course of schizophrenia, but they have reported inconsistent results. Specifically, some of these studies have demonstrated that FA reduction already exists at the onset of the illness, but others have found no decrease in FA until a decade into the illness course^11–13^.

Functional brain abnormalities have been extensively studied using functional MRI (fMRI), with some studies focusing on spontaneous neural activity of individual brain regions^14^ and others on functional connectivity between brain regions. Most studies finding altered functional connectivity between some brain regions n patients with schizophrenia have used seed-based analysis compared with those in healthy controls^15,16^. In contrast to the regions-of-interests approach in seed-based analysis, global functional connectivity density (gFCD) analysis can be used to measure the association of blood-oxygen-level-dependent (BOLD) signals between all voxels^17^. Some studies using gFCD analysis have observed hypoactivity in certain brain regions^18,19^. Nevertheless, how these abnormalities change over the course of schizophrenia has yet to be reported.

Inspired by electroencephalographic studies, the entropy of BOLD signal time series has recently been used to reflect the complexity of brain activity^20^. Among indicators of the BOLD signal entropy, sample entropy (SampEn) is one of the most commonly used, which measures the probability that two vectors that are near each other at some points will remain close at the next point^21^. Studies have observed altered SampEn of BOLD signals in some brain regions in patients with schizophrenia. However, further investigation is required to elucidate the meaning underlying this alteration^22^.

As to the order of progression in brain abnormalities, previous studies have primarily focused on single imaging modality or anatomic site. Luo et al. reported abnormality in functional connectivity both in first episode and in chronic schizophrenia patients, but abnormality in gray matter volume only in chronic schizophrenia patients^23^. Whether gray matter or white matter abnormalities occurred first in schizophrenia has almost never been investigated.

Accordingly, the present study explored whether and how brain abnormalities on structural and functional MRI scans in patients with schizophrenia progress across different periods of the illness course. We applied four analysis methods: VBM, FA, gFCD, and SampEn. A few previous studies showed correlation between VBM and FA in Alzheimer’s disease^24^, correlation between VBM and functional connectivity in normal adults^25^, correlation between FA and functional connectivity in healthy adults^26,27^, and correlation between entropy and functional connectivity in healthy adults^28^. However, correlation between these imaging modalities in schizophrenia has been rarely reported and some studies even found dissociation or segregation between structural and functional abnormalities in schizophrenia^29,30^. Since these four analysis methods are nearly independent from one another, delineating their progression patterns may elucidate the occurrence order of brain abnormalities in terms of structural/functional, gray matter/white matter, and individual brain regions/between brain regions.

## Methods

### Participants

We recruited patients with schizophrenia and healthy controls from the Department of Psychiatry at Taipei Veterans General Hospital, Taiwan. All patients with schizophrenia were evaluated by two board‐certified psychiatrists and diagnosed in accordance with the diagnostic criteria of the *Diagnostic and Statistical Manual of Mental Disorders, Fourth Edition Text Revision* (*DSM-IV-TR*). Diagnoses of schizophrenia were validated using the Mini‐International Neuropsychiatric Interview (MINI)^31^, and the Positive and Negative Syndrome Scale (PANSS) was used to evaluate psychotic symptoms^32^. Chlorpromazine (CPZ) equivalent dosages were computed in accordance with the American Psychiatric Association guidelines. All participants underwent the Wisconsin Card Sorting Test (WCST).

We recruited the controls from a healthy cohort and matched them with the patients with schizophrenia according to age and sex at a 2:1 control‐to‐patient ratio. The controls were evaluated by a trained research assistant using the MINI to exclude the presence of psychiatric disorders. Moreover, the controls had no personal or family history (first‐degree relatives) of psychiatric disorders. The exclusion criteria were as follows: having a history of head trauma or neurological disease, severe medical illness, or a history of substance abuse. The study was conducted in accordance with the Declaration of Helsinki and was approved by the Institutional Review Board of Taipei Veterans General Hospital and National Yang-Ming University.

### Image acquisition

All the fMRI experiments were performed at National Yang‐Ming University by using a 3.0 T Siemens MRI scanner (Siemens, Erlangen, Germany) equipped with a 12‐channel head coil, and the scanning was conducted in the morning. We measured T2*‐weighted images with BOLD contrast by using a 43‐slice gradient echo‐planar imaging (EPI) sequence with a repetition time (TR) of 2500 milliseconds, an echo time (TE) of 27 milliseconds, a field of view (FoV) of 200 mm, a flip angle of 77°, a matrix size of 64 × 64, and a voxel size of 3.44 × 3.44 × 3.40 mm. During each run, 200 EPI volume images were acquired along the anterior commissure–posterior commissure plane. We acquired high‐resolution structural 192‐slice T1 images by using a three-dimentional magnetization‐prepared rapid gradient echo sequence with a TR of 2530 milliseconds, TE of 3.5 milliseconds, TI of 1100 milliseconds, FoV of 256 mm, and flip angle of 7°. For each participant, the duration of each fMRI procedure was about 15 min.

### Image processing

We preprocessed the resting-state BOLD images using Data Processing Assistant for Resting‐State fMRI toolbox V4.3^33^ implemented in Matlab 2017b (MathWorks, Natick, MA). Tthe first five volumes were deleted to obtain steady-state tissue magnetization and the remaining 205 volumes were then corrected for slice-timing correction and head motion, realigned, co-registered to their own structural images, and normalized to the standard stereotaxic space of the Montreal Neurological Institute (MNI) standard space. Then the imaging data were resampled to a 3 mm^3^ voxel, and smoothed using a Gaussian kernel (FWHM = 6 mm). Before gFCD and SampEn analysis was performed, covariates of BOLD time series were regressed out, including the time courses of six head motions, white matter, and cerebrospinal fluid. We did not perform global signal regression to avoid introducing distortions in the time series data^34^. Physiological noise was minimized using band‐pass temporal filtering (0.01–0.08 Hz). All functional images with a framewise displacement of >0.9 mm were removed. The first five data points (12.5 s) in each BOLD time series were discarded because of the instability of the initial MRI scanning process; thus, 195 data points on each time series were used in the analyses.

### MRI analysis

#### VBM analysis

T1-weighted images were processed using the Diffeomorphic Anatomical Registration Through Exponentiated Lie Algebra (DARTEL) algorithm^35^ to create a study-specific template for the spatial normalization of the segmented images of each participant. After manual reorientation to place the anterior commissure at the origin of the 3-dimensional Montreal Neurological Institute (MNI) coordinate system, T1-weighted images were segmented into the following tissue classes: gray matter, white matter, and cerebrospinal fluid. Gray matter T1-weighted images were chosen to enter statistical analysis.

#### gFCD analysis

For each gray matter voxel, the corresponding gFCD was derived as the number of global functional connections, determined on the basis of Pearson’s correlation r between BOLD signals at that voxel and those in the remaining gray matter voxels, that exceeded an arbitrary threshold (r > 0.6)^11^.

#### DTI and FA analysis

To preprocess raw DTI images, we used the Functional Magnetic Resonance Imaging of the Brain (FMRIB) diffusion toolbox, included in the Functional Magnetic Resonance Imaging of the Brain Software Library (FSL)^36^. We then corrected the raw DTI images for the effects of head movement and eddy currents. Non-brain components of each image were removed with a brain extraction tool^37^. FA values were calculated by fitting a tensor model to the data at each voxel from these images. We then used the Tract-Based Spatial Statistics (TBSS, part of FSL) pipeline to register and normalize the FA images to the MNI standard space used to target the FMRIB58_FA standard-space image^38^. A mean FA skeleton was developed by creating and thinning a mean FA image to represent the centers of all tracts common to the group. An FA threshold of 0.2 was applied to exclude non-skeletal voxels. The aligned FA data were then projected onto this skeleton for each participant, and the results were subjected to further statistical analyses.

#### SampEn analysis

SampEn can be defined as the negative natural logarithm of the conditional probability that a dataset of length *N*, having repeated itself within a tolerance of *r* (similarity factor) for *m* points (pattern length), will also repeat itself for *m* + 1 points within r without allowing self-matches, when *N* approaches infinity and *r* approaches 0^21,39^. The SampEn of the BOLD signals was computed for all gray matter voxels to create a whole-brain SampEn parametric map.

### Statistical analysis

**Fig. 1:**
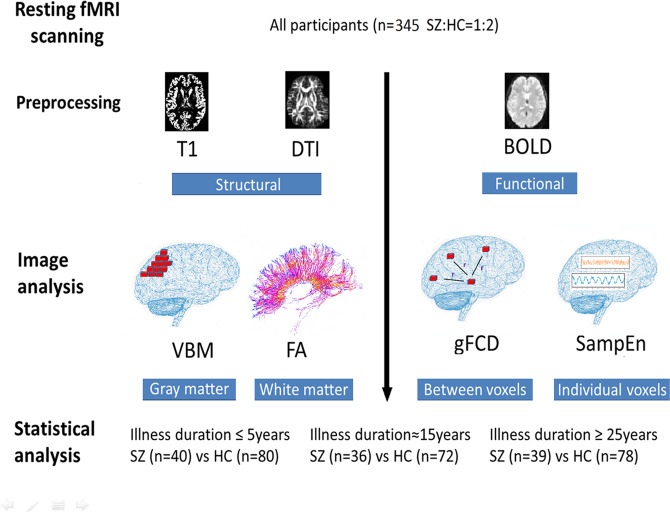
Flow chart of the analysis design. All participants underwent resting-state fMRI scanning. T1 images, diffusion tensor images (DTI), and blood-oxygen-level-dependent (BOLD) signals were preprocessed and then used for voxel-based morphometry (VBM), fractional anisotropy (FA), global functional connectivity (gFCD), and sample entropy (SampEn) analyses. The analysis results of the three groups of patients with schizophrenia (SZ) were compared with their healthy controls (HC), respectively.

The analysis flow is presented in Fig. 1. For group differences in demographic and clinical data, a *t*-test, Chi-square test, or ANOVA were conducted as appropriate by using IBM SPSS v22. The significance threshold was set at 0.05, two-tailed.

Regarding the VBM, gFCD, and SampEn analyses, group effects on brain regions were investigated through whole-brain voxel-wise comparisons of the preprocessed images of the patients with schizophrenia and those of the controls by using the general linear model (GLM) Analysis of Covariance (ANCOVA). Though sex and age were well matched, they were still used as covariates in the GLM ANCOVA analysis. Bonferroni correction for multiple comparisons was used to calculate between-group differences. For all voxel-wise analyses, significant brain clusters with peak co-ordinates in the MNI space were recorded only if the *p* value corrected for multiple comparisons using the family-wise error rate (*P*_FWE_) was < 0.05 at the cluster level and the number of voxels (*k*) in the cluster was ≥30. To evaluate the effects of illness duration and cumulative antipsychotics exposure (dose-years), we used them as confounding covariates, respectively, and repeated the GLM ANCOVA analyses.

For DTI-FA, we examined regional differences in whole-brain parametric mapping between the patients with schizophrenia and the controls by using the GLM. Based on 48 white matter track labels defined in the ICBM-DTI-81 atlas developed at Johns Hopkins University, we then determined the anatomical regions of interest^40^. The average FA of each white matter tract was calculated by overlaying the group-specific white matter skeleton and the ICBM-DTI-81 atlas with a specific white matter label. A *t*-test was used to assess between-group differences in FA. A *p* < 0.001 was used to assess between-group difference in FA measures (i.e., 0.05/48 tests).

## Results

### Participants

This study included 345 Han Chinese participants who were divided into three groups: 5-year, 15-year, and 25-year groups. The 5-year group consisted of 40 patients with schizophrenia (20 men and 20 women; mean age: 36.0 ± 11.8 years) and 80 healthy controls (39 men and 41 women; mean age: 37.0 ± 10.2 years). The 15-year group consisted of 36 patients with schizophrenia (12 men and 24 women; mean age: 44.4 ± 6.4 years) and 72 healthy controls (24 men and 48 women; mean age: 44.1 ± 7.1 years). Finally, the 25-year group consisted of 39 patients with schizophrenia (16 men and 23 women; mean age: 54.1 ± 8.5 years) and 78 healthy controls (36 men and 42 women; mean age: 55.2 ± 7.9 years). The participants’ demographic and clinical data are presented in Table 1. We found no significant within-group difference in age, sex, or handedness between the patients with schizophrenia and the controls in the 5-year, 15-year, or 25-year group. We also observed no significant difference in PANSS scores or CPZ equivalence between the patients with schizophrenia in the three groups, but illness onset age was higher in the 5-year group than in the 15-year and 25-year groups. Sixteen percent of all the recruited patients were being hospitalized in a psychiatric ward and 84% were outpatients. Ninety-two percent of the patients were in remission.

**Table 1:** Demographic and clinical characteristics.

variables	5-year group			15-year group			25-year group		
	SZ (*n* = 40)	HC (*n* = 80)	*p*	SZ (*n* = 36)	HC (*n* = 72)	*p*	SZ (*n* = 39)	HC (*n* = 78)	*p*
	Mean (SD)	Mean (SD)		Mean (SD)	Mean (SD)		Mean (SD)	Mean (SD)	
Age at scan(years)	36 (11.8)	37 (10.2)	0.94	44.4 (6.4)	44.1 (7.1)	0.93	54.1 (8.5)	55.2 (7.9)	0.92
Gender, female	20 (50%)	41 (52%)	0.92	21 (63%)	42 (64%)	0.92	23 (58%)	42 (60%)	0.89
Handedness, right	35 (97%)	77 (96%)	0.92	30 (97%)	60 (96%)	0.92	37 (96%)	67 (97%)	0.92
Age of onset	33 (11.8)			28.7 (7.9)			25 (7.3)		
Illness duration	2.54 (0.4)			15.7 (1.4)			29.7 (7.5)		
PANSS total	38.3 (9.2)			37.3 (9.2)			39.3 (9.2)		
PANSS positive	9.6 (3.2)			8.6 (3.3)			9.2 (3.5)		
PANSS negative	9.4 (5.2)			9.5 (5.7)			9.7 (5.1)		
PANSS general	20.3 (3.9)			21.3 (3.7)			22.5 (3.8)		
CPZ equivalence	491 (390)			425 (312)			398 (278)		

*SZ* patients with schizophrenia; *HC* healthy controls; *PANSS* Positive and Negative Syndrome Scale; *CPZ* chlorpromazine.

*p* < 0.05.

### VBM analysis

Structural brain abnormalities in gray matter density determined in the three groups through VBM analysis are presented in Table 2. In the 5-year group, we observed no significant difference in gray matter density in any brain region between the patients with schizophrenia and the healthy controls. However, in the 15-year group, we noted significant differences in gray matter density in several brain regions, namely the left anterior cingulum, right insula and right lingual gyrus between the patients with schizophrenia and the healthy controls (all *P*_FWE_ < 0.05). Compared with the 15-year group, the 25-year group had more brain regions, namely the left insula, right cerebellum, left middle cingulum, right middle temporal gyrus, left superior middle frontal gyrus, that exhibited significant differences in gray matter density between the patients with schizophrenia and the healthy controls (all *P*_FWE_ < 0.05).

**Table 2:** Brain Regions with Significant Differences in voxel-based morphometry (VBM), fractional anisotropy (FA), global functional connectivity (gFCD), and sample entropy (SampEn) Between the three Groups of Patients with Schizophrenia and the Healthy Controls.

		Brain regions	Co-ordinates of maximal voxel	k (voxel number)	Peak t	direction
5-year group	VBM	Nil	Nil	Nil	Nil	HC > SZ
	FA	No significant difference in any of the 48 white matter tracts				
	gFCD	Nil	Nil	Nil	Nil	
	SampEn	L putamen	−21 3 3	62	4.64	HC > SZ
		R putamen	15 9 −6	65	3.82	HC > SZ
15-year group	VBM	L anterior cingulum	−2 30 −3	194	7.6	HC > SZ
		R insula	45 15 0	213	7.52	HC > SZ
		R lingual gyrus	21 −65 −6	34	6.45	HC > SZ
	FA	No significant difference in any of the 48 white matter tracts				
	gFCD	L caudate	−20 18 8	84	5.01	HC > SZ
		R inferior temporal gyrus	45 −25 −26	78	4.89	HC > SZ
		L inferior temporal pole	−36 −9 −42	76	4.76	HC > SZ
	SampEn	R putamen	18 15 −3	63	6.73	HC > SZ
		R lingual gyrus	9 −63 −3	46	4.34	HC < SZ
25-year group	VBM	L insula	−42 14 −3	7000	8.59	HC > SZ
		R cerebellum	5 −59 −59	320	6.77	HC > SZ
		L middle cingulum	−5 −14 36	560	5.72	HC > SZ
		R middle temporal gyrus	62 0 −15	84	5.34	HC > SZ
		L superior middle frontal gyrus	−2 48 23	172	5.32	HC > SZ
		R insula	35 −5 12	145	4.97	HC > SZ
	FA	All 48 white matter tracts other than 4 ones showed significant difference (See Supplemental Fig. 1)				HC > SZ
	gFCD	L caudate	−15 15 9	287	4.27	HC > SZ
		R inferior temporal gyrus	48 −24 −24	60	4.15	HC > SZ
		L inferior temporal gyrus	−54 −24 −24	53	4.07	HC > SZ
		R superior frontal gyrus	15 57 39	63	3.91	HC > SZ
		R cerebellum crus	30 −84 −48	83	3.71	HC > SZ
		L cerebellum crus	−24 −81 −45	57	3.51	HC > SZ
	SampEn	R putamen	18 15 −3	73	4.54	HC > SZ
		R precuneus	6 −57 27	78	4.14	HC < SZ

*p* < 0.05, corrected for multiple comparison using family-wise error at cluster level; only clusters with *k* ≥ 30 were reported.

### FA analysis

Structural brain abnormalities based on FA in the three groups are provided in Table 2 and Supplemental Fig. 1. In the 5-year group and 15-year groups, we observed no significant difference in FA values in any brain region between the patients with schizophrenia and the healthy controls. However, in the 25-year group, we observed that the patients with schizophrenia had significantly lower FA values than the healthy controls in all except 4 of the 48 white matter tracts.

### gFCD analysis

Functional brain abnormalities based on gFCD analysis in the three groups are presented in Table 2. In the 5-year group, we observed no significant difference in gFCD in any brain region between the patients with schizophrenia and the healthy controls. In the 15-year group, we noted significant differences in gFCD between the patients with schizophrenia and the controls in few brain regions, namely the left caudate, right inferior temporal gyrus, and left inferior temporal pole (all *P*_FWE_ < 0.05). Compared with the 15-year group, the 25-year group had more brain regions, namely the left caudate, right and left inferior temporal gyrus, right superior frontal gyrus, and right and left cerebellum crus, that exhibited significant differences in gFCD between the patients with schizophrenia and the healhty controls (all *P*_FWE_ < 0.05).

### SampEn analysis

Functional brain abnormalities based on SampEn analysis in the three groups are presented in Table 2. In the 5-year group, we observed significant differences in SampEn values in the right and left putamen between the patients with schizophrenia and the healhty controls. In the 15-year group, we noted significant differences in SampEn values in the right putamen and right ligual gyrus between the patients with schizophrenia and the healthy controls. Finally, in the 25-year group, we observed significant differences in SampEn values in the right putamen and right precuneus between the patients with schizophrenia and the healthy controls (all *P*_FWE_ < 0.05).

### Structural and functional abnormalities across different illness durations

**Fig. 2:**
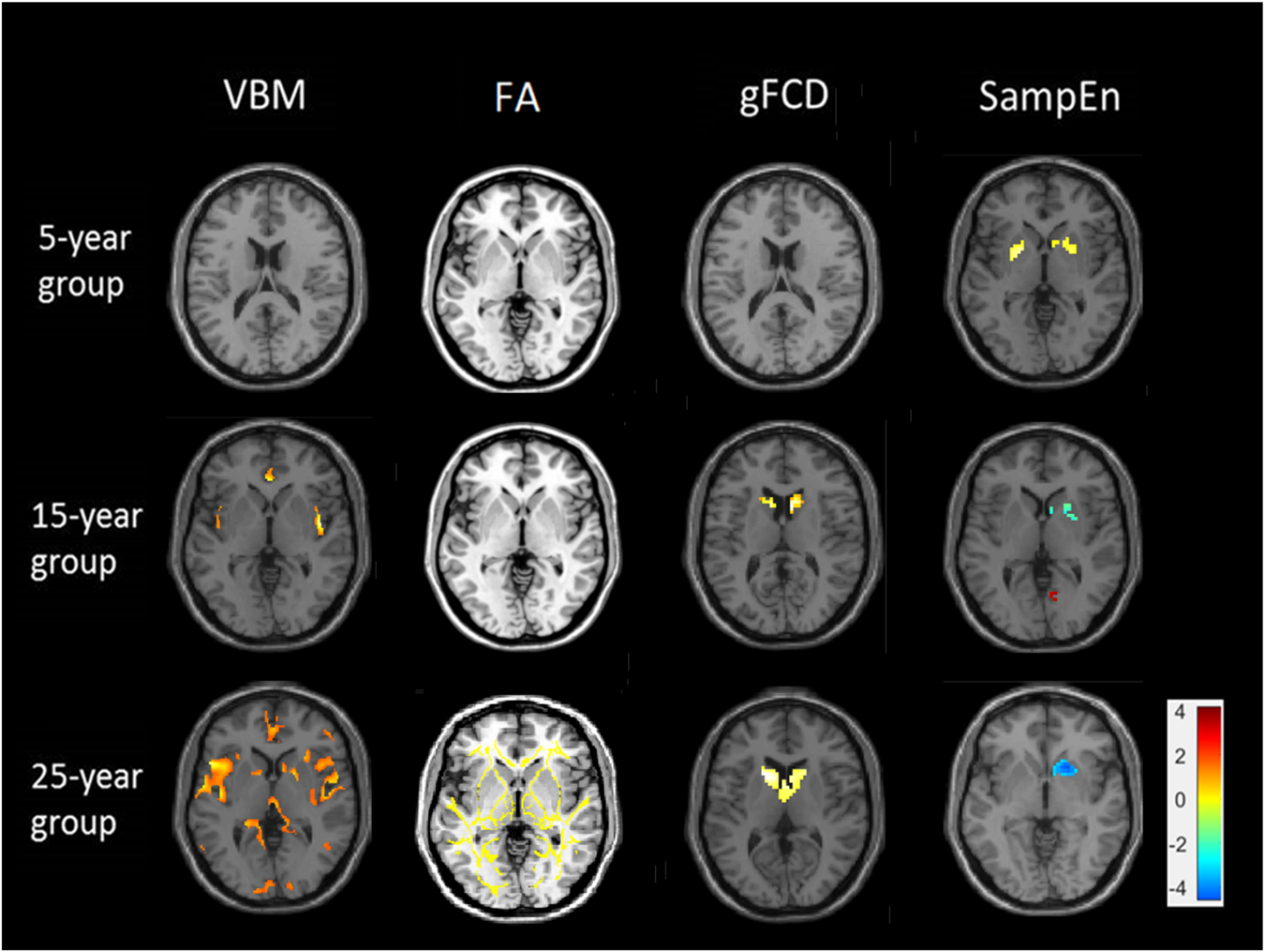
Brain regions with significant difference in voxel-based morphometry (VBM), fractional anisotropy (FA), global functional connectivity (gFCD), and sample entropy (SampEn) analyses between the patients with schizophrenia and the healthy controls. A positive *t* value represent healthy controls have a larger values than the patients with schizophrenia in the analyses.

**Fig. 3:**
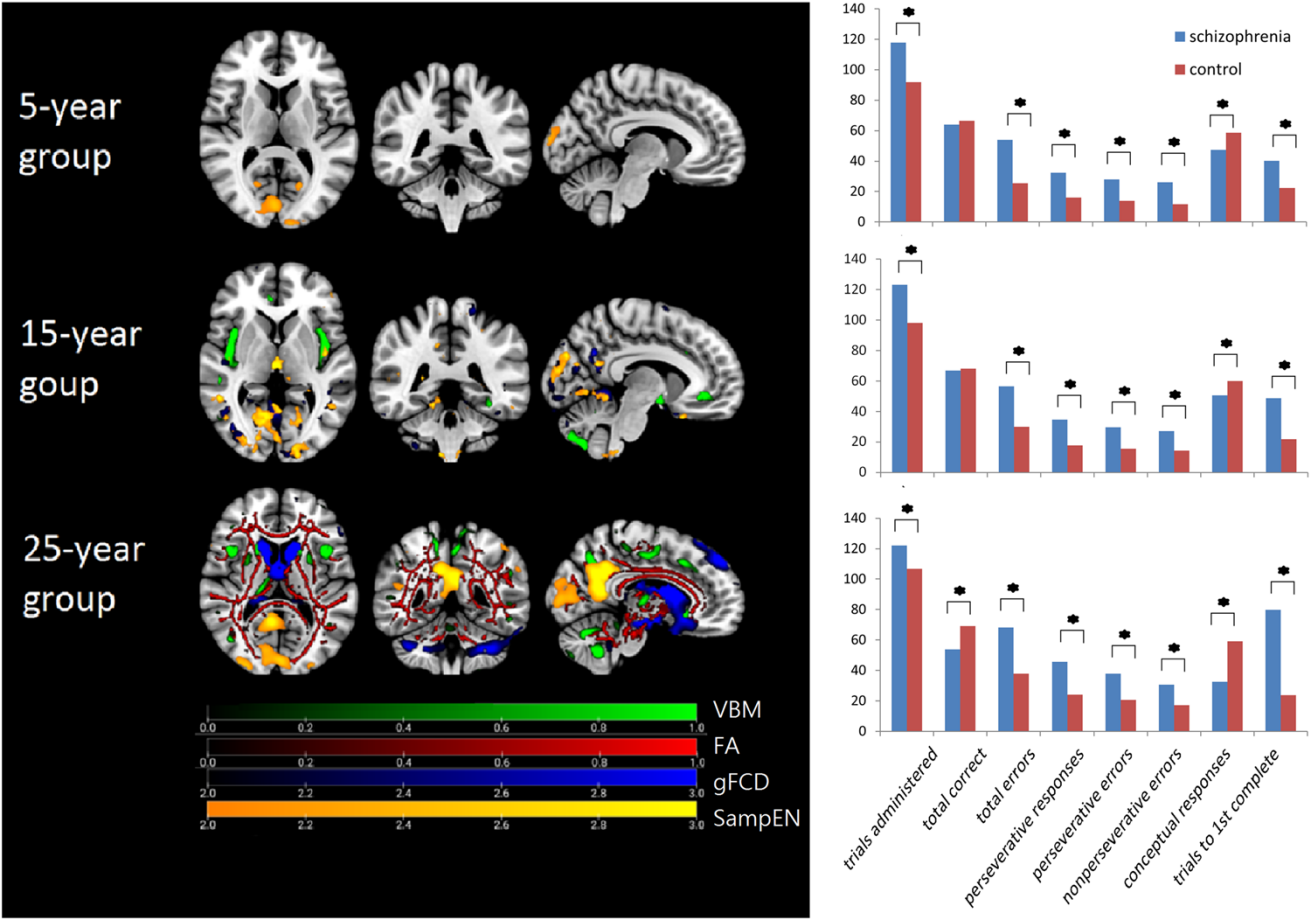
Progression patterns of brain abnormalities and WCST deficits. Left: the results of voxel-based morphometry (VBM), fractional anisotropy (FA), global functional connectivity (gFCD), and sample entropy (SampEn) analyses overlaid on the same template. The colored areas represent brain regions with significant difference between the patients with schizophrenia and the healthy controls. Right: comparison of WCST scores between the patients and the controls. The color bars of the left figure do not reflect statistical test values. The asterisks of the right figure represent *p* > 0.05.

Figure 2 shows the brain regions that differed significantly, as determined in the four analysis methods, between the patients with schizophrenia and the healthy controls in all the three groups. Figure 3 depicts composite images of the results obtained from the four analysis methods, which clearly shows the progressive pattern of structural and functional change in the brain of the patients with schizophrenia.

### Effects of age illness onset and cumulative exposure to antipsychotics on brain abnormalities

Combining all the patients with schizophrenia into one group, we analyzed the correlation of age illness onset with gray matter density, FA, gFCD, and SampEn; the results revealed no significant correlation in any brain region. Additionally, by using dose-years as the total amount of antipsychotics use, we found no brain region with a significant correlation between accumulative exposure to antipsychotics and brain abnormalities (as determined from the four analyses) in the patients with schizophrenia.

### WCST and brain abnormalities

There were eight items in our WCST: trials administered, total correct responses, total errors, perseverative responses, perseverative errors, non-perseverative errors, conceptual responses, and total trials to complete the first category. In the 5-year group and the 15-year group, The WCST results showed significant differences between the patients with schizophrenia and the healthy controls in seven of eight itmes, except for total correct responses. In the 25-year group, all the eight items showed significant differences. Compared to the imaging analyses, the WCST deficits had existed in earlier periods of schizophrenia and persisted into the third decade of the illness course, which was consistent with the SampEn analysis and inconsistent with the VBM, gFCD, and DTI analyses.

## Discussion

To our knowledge, this is the first study to determine the chronological pattern of structural and functional brain abnormalities occurring across three decades of illness course of schizophrenia, by using four types of imaging analysis and three groups of participants with different illness durations of schizophrenia. In the 5-year group, brain abnormalities appeared only in the putamen with decreased SampEn. In the 15-year group, the left insula and the right cingulate gyrus exhibited reduced gray matter density, the left caudate and left inferior temporal gyrus exhibited decreased gFCD, and the right putamen exhibited decreased SampEn and the right lingual gyrus exhibited increased SampEn; however, no deficit in white matter tract integrity was observed in this group. In the 25-year group, we observed more extensive brain regions exhibiting significant changes between the patients and the healthy controls compared with the other groups. Reduced gray matter density was found in the insula, left cingulate gyrus, right temporal gyrus, left superior frontal gyrus, and right cerebellum; integrity deficits occurred in almost all white matter tracts; reduced gFCD was observed in the left caudate, inferior temporal gyrus, right superior frontal gyrus, and the cerebellum crus. Decreased SampEn values, however, occurred only in the right putamen and increased SampEn occurred only in the right precuneus. Cognitive deficits measured with the WCST showed a corresponding pattern of progression only to the SampEn analysis in our study.

### Progression of brain abnormalities throughout the illness course

Consistent with other reports, this study demonstrated that brain abnormalities occurred in limited brain regions in the early stages of schizophrenia and gradually spread in later years^41–44^. However, in contrast to previous studies, we stratified the patients with schizophrenia into three groups according to illness duration and demonstrated that brain abnormalities continued to progress into the third decade of illness. Because we compared the three patient groups with three groups of age-matched healthy controls, these progressive brain abnormalities cannot be attributed to aging-related changes in brain structure and function.

Although most studies using VBM analysis have reported gray matter density reduction in patients in the early stages of schizophrenia, some researchers revealed no such results^45,46^. Our study corroborated the latter’s finding and revealed no difference in gray matter density between the brain regions of patients diagnosed with schizophrenia for less than 5 years and those of the controls. A possible reason may be patients with schizophrenia in our study had lower PANSS scores, which means less severe in symptoms, than previous studies^14,47,48^. We observed reduction in gray matter density in the brains of the patients diagnosed with schizophrenia for more than 5 years, and this reduction continued to progress until the third decade of the illness course. In line with previous studies, we found regional gray matter abnormalities in in the putamen^49^, the insula^50^, and the temporal and frontal cortex^51^, but our studies furthermore revealed that the subcortical abnormalities predate cortical ones^42^.

In previous studies, reduced FA value has been observed in the white matter tracts in patients with schizophrenia patients, and this reduction occurred as early as the first episode, but subsequent studies have demonstrated that FA reduction emerged only after about 10 years after diagnosis^12,13,52^. The present study extended the illness duration for onset of white matter abnormalities into 15 years after diagnosis and demonstrated by 25 years after diagnosis, almost all white matter tracts exhibited FA reduction. Combined with our VBM analysis, we also found, as to structural abnormalities, gray matter is involved earlier than white matter, which was rarely mentioned before.

Most studies on functional connectivity deficits in patients with schizophrenia have used seed-based analysis, which is subject to seed selection bias^53^. In contrast, global functional connectivity analysis is theory-free and the results of several studies using this method revealed a reduction in global functional connectivity in some brain regions of patients with schizophrenia^54,55^. However, whether the reduction increased over the course of the illness course is unclear. The present study is the first to demonstrate that gFCD reduction occurred in the second decade and progressed into the third decade after a diagnosis of schizophrenia. Interestingly, unlike previous reports^56,57^, the progressive pattern of gFCD abnormalities was relatively similar to VBM in our study, which is not unreasonable since it is hard for reduced gray matter density to maintain existing functional connection.

In contrast to connectivity analysis, SampeEn measures the complexity of BOLD activity in individual brain regions^22,39^. Reduced SampEn in some brain regions has been noted in patients with schizophrenia^58^, but no study has reported how it changes throughout the course of the illness. We demonstrated that SampEn reduction occurred in the early statge of schizophrenia and continue to affect almost the same brain regions in later stages of the illness. One explanation is that SampEn may be a trait marker or endophenotype, rather than a state marker, of schizophrenia^59^. In contrast to phenotypes, an endophenotype will not change with the severity of symptomatology, remained stable throughout the illness course and has heritability, such as P50 abnormalities of audio-evoked potential in electroencephalogram in schizophrenia^60^. Hager et al. used first-degree relatives as probands to show the possibility of multi-scale entropy of BOLD signals as biomarkers for psychosis^61^. Since we did not recruit family members of the patients with schizophrenia as participants, further research is needed to confirm our inference.

### Chronological pattern of brain abnormality progression

Since Kraepelin’s identification of the disorder, schizophrenia has been construed as a neurodegenerative disorder^62^, but how the brain degenerates over the course of the illness remained undetermined. By using four brain MRI analyses, we demonstrated that functional abnormalities initially occurs in individual brain region in the early stages of schizophrenia, abnormalities in functional connectivity between brain regions and gray matter abnormalities ensue in the middle stages, and structural connectivity abnormalities manifesting with the disintegration of white matter tracts emerge during the later stages. Brain abnormalities occur in limited areas in the early stages of schizophrenia and progress into more areas in later stages.

Age of onset has been shown to correlate with illness severity of schizophrenia and may thus affect the extent of brain abnormalities^63^, but in this study we did not find significant correlations between age of onset and the findings in four analysis methods we used. Although previous studies reported exposure to antipsychotics may contribute to brain abnormalities found in patients with schizophrenia^64^, no such significant correlations were noted in ours. Therefore, brain abnormalities observed in our study could not be attributed to differences in age of onset of the illness or length and amount of exposure to antipsychotics.

Previous studies obtained inconsistent findings about correlation of cognitive deficits and structural or functional brain abnormalities in schizophrenia^65–67^, and some brain regions were reported to be implicated with the WCST deficits in the patients^68,69^. Among our four imaging analyses, only SampEn showed a corresponding pattern of brain abnormalities to the WCST results. The SampEn analysis also showed the earilest brain abnormalities among the four analysis modalities. Accordingly, our study suggests BOLD signal entropy abnormalities may be a more sensitive and consistent neurological correlates than other structural or functional analysis modalities for investigating brain abnormalities in schizophrenia.

### Limitations

Our study has several limitations. First, although our results demonstrated the progression of brain abnormalities over the course of schizophrenia, this was a cross-sectional study, that placed patients with schizophrenia into three groups depending on the length of their diagnosis. Therefore, longitudinal changes could only be indirectly inferred. However, since a longitudinal study of patients throughout the course of schizophrenia is not feasible, a cross-sectional one using respective age-matched control groups is a reasonable alternative. Second, we did not perform global signal regression to prevent BOLD signal distortion and its effects on functional connectivity and entropy estimates may differ between the patients with schizophrenia and the healthy controls^70^. Third, although the SampEn changes remained stationary throughout the course of the illness in our study, a more comprehensive profile of brain signal complexity may be obtained through multi-scale entropy. Finally, our study used resting-state fMRI, but some brain abnormalities may manifest only during activation of brain regions, which may be detected only by using task-related fMRI studies.

### Conclusion

Using structural and functional brain MRI analysis, we compared brain abnormalities between three groups of patients with schizophrenia patients with varying illness durations and their corresponding age and sex-matched healthy controls. Our findings represent a novel approach to studying the progression of brain abnormalities in patients with schizophrenia. On a broader scale, analyzing the chronological pattern of brain changes in patients with schizophrenia may increase our understanding about how brain abnormalities evolve in patients with schizophrenia.

## Supplementary information

Supplemental Figure 1
