# Supplementary material for: Progressive brain abnormalities in schizophrenia across different illness periods: a structural and functional MRI study
Source: Schizophrenia (Heidelb). 2023 Jan 5;9(1):2. doi: 10.1038/s41537-022-00328-7 (PMC9816110; doi:10.1038/s41537-022-00328-7)
Supplement: Supplementary file 1 — Supplemental Figure 1 [file 41537_2022_328_MOESM1_ESM.docx]

**Supplemental Figure 1** Differences Between the 25-year group of Patients with Schizophrenia and the Healthy Controls in Fractional Anisotropy in 48 White Matter Tracts.

| **No.** | **White Matter Tract** | **HC** | **SZ** | ***t*** | ***P*** | **Direction** |
| --- | --- | --- | --- | --- | --- | --- |
| 1 | Mid. cerebellar peduncle | 0.53 ± 0.13 | 0.39 ± 0.12 | 4.81 | < 0.001 | HC > SZ |
| 2 | Pontine crossing tract | 0.56 ± 0.12 | 0.41 ± 0.11 | 4.49 | < 0.001 | HC > SZ |
| 3 | Genu of corpus callosum | 0.60 ± 0.02 | 0.33 ± 0.08 | 5.0 | < 0.001 | HC > SZ |
| 4 | Body of corpus callosum | 0.56 ± 0.02 | 0.35 ± 0.09 | 4.81 | < 0.001 | HC > SZ |
| 5 | Splenium of corpus callosum | 0.47 ± 0.02 | 0.33 ± 0.11 | 3.9 | < 0.001 | HC > SZ |
| 6 | Fornix column and body of fornix | 0.46 ± 0.06 | 0.41 ± 0.08 | 3.21 | n.s. |  |
| 7 | Corticospinal tract R | 0.49 ± 0.10 | 0.45 ± 0.13 | 3.3 | n.s. |  |
| 8 | Corticospinal tract L | 0.48 ± 0.10 | 0.45 ± 0.11 | 3.21 | n.s. |  |
| 9 | Medial lemniscus R | 0.53 ± 0.16 | 0.40 ± 0.16 | 4.17 | < 0.001 | HC > SZ |
| 10 | Medial lemniscus L | 0.58 ± 0.18 | 0.38 ± 0.16 | 5.74 | < 0.001 | HC > SZ |
| 11 | Inf. cerebellar peduncle R | 0.42 ± 0.13 | 0.26 ± 0.14 | 4.91 | < 0.001 | HC > SZ |
| 12 | Inf. cerebellar peduncle L | 0.39 ± 0.16 | 0.23 ± 0.16 | 4.49 | < 0.001 | HC > SZ |
| 13 | Sup. cerebellar peduncle R | 0.58 ± 0.06 | 0.43 ± 0.11 | 4.93 | < 0.001 | HC > SZ |
| 14 | Sup. cerebellar peduncle L | 0.55 ± 0.06 | 0.43 ± 0.09 | 3.92 | < 0.001 | HC > SZ |
| 15 | Cerebral peduncle R | 0.62 ± 0.02 | 0.53 ± 0.12 | 4.21 | < 0.001 | HC > SZ |
| 16 | Cerebral peduncle L | 0.58 ± 0.02 | 0.50 ± 0.10 | 4.7 | < 0.001 | HC > SZ |
| 17 | Ant. limb of int. capsule R | 0.51 ± 0.02 | 0.46 ± 0.08 | 3.66 | < 0.001 | HC > SZ |
| 18 | Ant. Limb of Int. Capsule L | 0.52 ± 0.02 | 0.46 ± 0.08 | 3.81 | < 0.001 | HC > SZ |
| 19 | Post. limb of int. capsule R | 0.64 ± 0.02 | 0.48 ± 0.12 | 5.53 | < 0.001 | HC > SZ |
| 20 | Post. limb of int. capsule L | 0.52 ± 0.01 | 0.47 ± 0.08 | 4.04 | < 0.001 | HC > SZ |
| 21 | Retrolenticular R | 0.58 ± 0.02 | 0.38 ± 0.08 | 6.8 | < 0.001 | HC > SZ |
| 22 | Retrolenticular L | 0.46 ± 0.02 | 0.39 ± 0.07 | 3.51 | < 0.001 | HC > SZ |
| 23 | Ant. corona radiata R | 0.51 ± 0.02 | 0.37 ± 0.04 | 5.06 | < 0.001 | HC > SZ |
| 24 | Ant. corona radiata L | 0.48 ± 0.03 | 0.38 ± 0.05 | 4.45 | < 0.001 | HC > SZ |
| 25 | Sup. corona radiata R | 0.51 ± 0.02 | 0.43 ± 0.06 | 4.15 | < 0.001 | HC > SZ |
| 26 | Sup. corona radiata L | 0.49± 0.02 | 0.40 ± 0.06 | 3.87 | < 0.001 | HC > SZ |
| 27 | Pos. corona radiata R | 0.42 ± 0.02 | 0.40 ± 0.06 | 0.82 | n.s. |  |
| 28 | Pos. corona radiata L | 0.52± 0.02 | 0.41 ± 0.06 | 3.97 | < 0.001 | HC > SZ |
| 29 | Post. thalamic radiation R | 0.55 ± 0.04 | 0.45 ± 0.07 | 4.16 | < 0.001 | HC > SZ |
| 30 | Post. thalamic radiation L | 0.53 ± 0.05 | 0.40 ± 0.09 | 4.22 | < 0.001 | HC > SZ |
| 31 | Sagittal stratum R | 0.45 ± 0.07 | 0.34 ± 0.13 | 4.8 | < 0.001 | HC > SZ |
| 32 | Sagittal stratum L | 0.49 ± 0.06 | 0.32 ± 0.10 | 5.01 | < 0.001 | HC > SZ |
| 33 | External capsule R | 0.49 ± 0.02 | 0.38± 0.06 | 4.69 | < 0.001 | HC > SZ |
| 34 | External capsule L | 0.47 ± 0.01 | 0.34 ± 0.05 | 4.36 | < 0.001 | HC > SZ |
| 35 | Cingulum cingulate gyrus R | 0.54 ± 0.03 | 0.40 ± 0.08 | 4.71 | < 0.001 | HC > SZ |
| 36 | Cingulum cingulate gyrus L | 0.46 ± 0.02 | 0.34 ± 0.05 | 4.96 | < 0.001 | HC > SZ |
| 37 | Cingulum hippocampus R | 0.44 ± 0.03 | 0.32 ± 0.07 | 4.54 | < 0.001 | HC > SZ |
| 38 | Cingulum hippocampus L | 0.61 ± 0.04 | 0.29 ± 0.08 | 5.78 | < 0.001 | HC > SZ |
| 39 | Fornix cres stria terminalis R | 0.50 ± 0.02 | 0.42 ± 0.09 | 5.25 | < 0.001 | HC > SZ |
| 40 | Fornix cres stria terminalis L | 0.53 ± 0.02 | 0.37 ± 0.08 | 5.42 | < 0.001 | HC > SZ |
| 41 | Sup. longitudinal fasciculus R | 0.56 ± 0.02 | 0.43 ± 0.07 | 4.56 | < 0.001 | HC > SZ |
| 42 | Sup. longitudinal fasciculus L | 0.53 ± 0.02 | 0.41 ± 0.06 | 4.28 | < 0.001 | HC > SZ |
| 43 | Sup. fronto occipital fasciculus R | 0.47 ± 0.03 | 0.32 ± 0.09 | 5.55 | < 0.001 | HC > SZ |
| 44 | Sup. fronto occipital fasciculus L | 0.48 ± 0.03 | 0.32 ± 0.07 | 4.47 | < 0.001 | HC > SZ |
| 45 | Uncinate fasciculus R | 0.51 ± 0.03 | 0.37 ± 0.10 | 4.99 | < 0.001 | HC > SZ |
| 46 | Uncinate fasciculus L | 0.48 ± 0.03 | 0.34 ± 0.07 | 4.09 | < 0.001 | HC > SZ |
| 47 | Tapetum R | 0.52 ± 0.04 | 0.41 ± 0.07 | 4.74 | < 0.001 | HC > SZ |
| 48 | Tapetum L | 0.55 ± 0.03 | 0.47 ± 0.09 | 3.42 | < 0.001 | HC > SZ |

n.s., non-significant. All results had *P* < .001 using Bonferroni corrections for multiple comparisons (i.e., 0.05 divided by 48 tests)
